# Eviction-driven infanticide and sexually selected adoption and infanticide in a neotropical parrot

**DOI:** 10.1073/pnas.2317305121

**Published:** 2024-05-06

**Authors:** Steven R. Beissinger, Karl S. Berg

**Affiliations:** ^a^Department of Environmental Science, Policy & Management, University of California, Berkeley, CA 94720; ^b^Museum of Vertebrate Zoology, University of California, Berkeley, CA 94720; ^c^School of Integrative Biological and Chemical Sciences, University of Texas Rio Grande Valley, Brownsville, TX 78520

**Keywords:** adoption, infanticide, sexual selection, sexual conflict, sexual cooperation

## Abstract

Infanticide and adoption have been attributed to sexual selection, where an individual later reproduces with the parent whose offspring it killed or adopted. While sexually selected infanticide is well known, evidence for sexually selected adoption is anecdotal. We report on both behaviors at 346 nests over 27 y in green-rumped parrotlets (*Forpus passerinus*) in Venezuela. Parrotlets are monogamous with long-term pair bonds, exhibit a strongly male-biased adult sex ratio, and nest in cavities that are in short supply, creating intense competition for nest sites and mates. Infanticide attacks occurred at 256 nests in two distinct contexts: 1) Attacks were primarily committed by nonbreeding pairs (69%) attempting to evict parents from the cavity. Infanticide attacks per nest were positively correlated with population size and evicting pairs never adopted abandoned offspring. Competition for limited nest sites was a primary cause of eviction-driven infanticide, and 2) attacks occurred less frequently at nests where one mate died (31%), was perpetrated primarily by stepparents of both sexes, and was independent of population size. Thus, within a single species and mating system, infanticide occurred in multiple contexts due to multiple drivers. Nevertheless, 48% of stepparents of both sexes adopted offspring, and another 23% of stepfathers exhibited both infanticide and long-term care. Stepfathers were often young males who subsequently nested with widows, reaching earlier ages of first breeding than competitors and demonstrating sexually selected adoption. Adoption and infanticide conferred similar fitness benefits to stepfathers and appeared to be equivalent strategies driven by limited breeding opportunities, male-biased sex ratios, and long-term monogamy.

## Significance

Infanticide and adoption are puzzling forms of sexual conflict and cooperation, respectively. However, both may be explained by sexual selection, where an individual later reproduces with the parent whose offspring it killed or adopted. While sexually selected infanticide is well known, evidence for sexually selected adoption is anecdotal. Our long-term study of a parrotlet in Venezuela found infanticide attacks were mostly enacted by nonbreeding pairs attempting to evict parents from their nests to usurp the cavity. Infanticide attacks occurred less often at nests where a parent died, and adoption by stepparents was as common as infanticide. Becoming an adoptive stepfather led to future nesting with the widow and an earlier age of first breeding than competitors, demonstrating sexually selected adoption.

Infanticide and adoption in animal and human societies have fascinated evolutionary biologists as puzzling forms of sexual conflict (1–3) and cooperation (4), respectively. A stepparent that acquired a mate with preexisting dependent offspring, or an individual who has immigrated into a social group with unrelated, vulnerable offspring, must choose between infanticide via direct aggression or neglect of offspring (5) and adoption, defined broadly as a spectrum of behavior ranging from tolerance of the offspring to active parental investment (6, 7). Yet, when infanticide and adoption occur in this context, similar adaptive explanations have been proffered for both (7).

Sexual selection has long been proposed to explain infanticide by males in group-living mammals in which one or a few males compete to monopolize access to multiple females (1, 6, 8) and in birds with biparental care that have suffered the death of a parent (9, 10). Infanticide in this context is primarily viewed as a strategy to shorten the time to the next reproductive attempt for males in mammals (11–13) and for both sexes in birds when sexual selection is defined broadly to include female–female competition (14–16). Similarly, infanticide may monopolize access to mates by protecting the dominance status in mammals with social hierarchies (17–19).

Sexual selection may also drive adoptions by avian stepparents and mammalian males immigrating into groups if it functions as a mating effort to promote the opportunity for the stepparent or killer to reproduce later with the widowed parent (6, 7, 10). Adoption requires restraint from the stepparent (10), vigilance behaviors by the widowed parent to defend the offspring from the stepparent until it has developed sufficiently to be relatively safe (20–22), or both. Adoption by avian stepparents appears more common than infanticide (6, 23, 24) and is predicted for species that exhibit skewed sex ratios, produce multiple broods in a breeding season, and have pair bonds that persist across breeding seasons (10). Nevertheless, direct evidence in support of sexually selected adoption in animals and humans—where the stepparent later reproduces with the widowed parent—is primarily anecdotal (6, 7).

Infanticide in birds and mammals also occurs in another context unrelated to mate loss or social hierarchies, driven instead by advantages accrued when breeding opportunities are limited by intense competition for territories, nest sites, and burrows required for reproduction (5, 25, 26). Infanticide in this context occurs when conspecifics attempt to evict a parent or pair caring for offspring, killing the offspring before or after the parents have been evicted. In birds with biparental care, which represents over 80% of avian species (27), eviction-driven infanticide appears to be rare (9, 25, 28) but may occur when nest sites are limited (10). Thus, eviction-driven infanticide should be density-dependent and facultative, depending on the relationship between competitors (e.g., adult population size) and the availability of resources (e.g., nest sites). In contrast, when it is preceded by the death of a parent in socially monogamous species, sexually selected infanticide by stepparents should occur independent of population density and instead depend on the mortality rate of parents during breeding, which would most likely be driven by predator density and density-independent factors (e.g., disease and weather). Nevertheless, it can be difficult to distinguish eviction-driven infanticide motivated by acquiring resources from sexually selected infanticide enacted to garner mating opportunities if the takeover of a territory or nest site includes the acquisition of potential mates (29–31), or vice versa.

Here, we document eviction-driven infanticide, and both sexually selected adoption and infanticide in the green-rumped parrotlet (*Forpus passerinus*) from events enacted by males and females at 346 nests over 27 y of population monitoring as well as during a mate-removal experiment that allowed us to observe the process of mate replacement at nests with widows. This socially monogamous parrot is small (25 to 30 g), inhabits tropical savannas, feeds primarily on seeds dispersed over large undefended areas, and nests in cavities that are vigorously defended and often in short supply (32, 33). It exhibits strong sexual dichromatism but no sex differences in body size, and has a strong male-biased adult sex ratio (due to low local survival of juvenile females) that varies little across years (median = 1.5 males per female), with many males unable to find mates (34–36). Parrotlet reproduction is characterized by highly stable pair bonds, courtship feeding by males and mate guarding throughout the breeding season, low extra-pair paternity, strong nest site fidelity, slow embryonic development, and highly altricial nestlings that require long periods for development (~50 d), and multiple brooding within discrete breeding seasons (37–39). Killing of eggs and nestlings by unrelated parrotlet adults has been previously documented (33, 40).

We examine the contexts and ecology of infanticide and adoption in parrotlets, concentrating on prevalence, timing, and relationships with adult population size. We describe the process of acquiring a stepparent at nests with widowed parents, and the social status and ages of stepparents, attackers, and adopters. Finally, we quantify the key fitness consequences of infanticide and adoption. We found that individuals who evict parents from nest sites and kill their offspring can later nest in the coveted cavity, and that becoming a stepfather—either adoptive or infanticidal—leads to an earlier age of first breeding than competitors. However, infanticidal and adoptive stepfathers did not differ in the probability of nesting subsequently with the widow whose offspring they killed or adopted, or in the number of offspring produced throughout their lifetime with the widow.

## Results

### Contexts and Ecology of Infanticide.

**Fig. 1.**
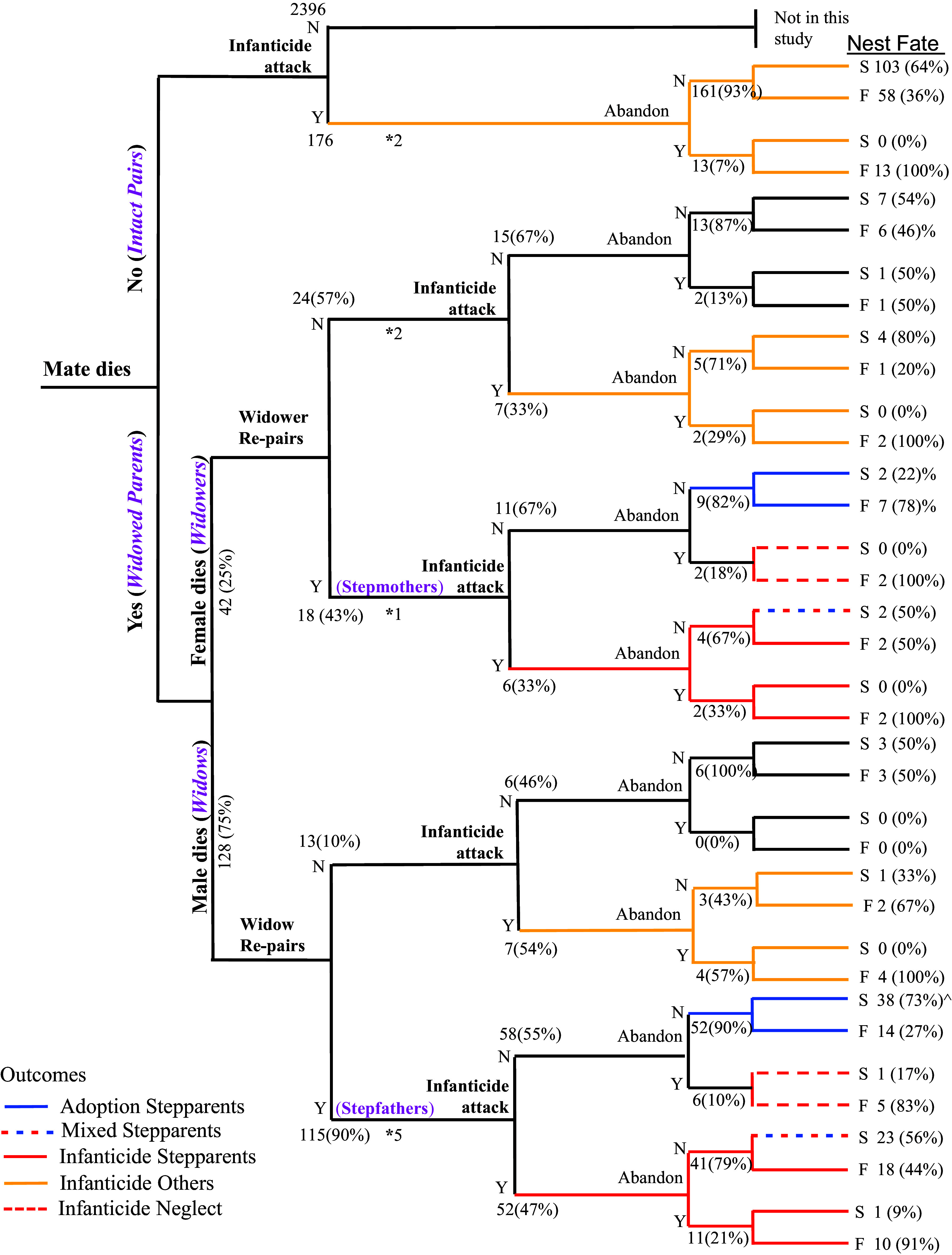
Tree of mate loss, infanticide attacks, re-pairing, nest abandonment, and adoption events leading to the fate of 346 nests of the green-rumped parrotlet featured in this study. Two distinct contexts of infanticide attacks are distinguished: 1) In the absence of a mate’s death (Intact Pairs), attacks were enacted by other parrotlet individuals or pairs (*n* = 176). Of the 2,742 nesting attempts monitored, 2,396 (90.7%) did not experience infanticide or a mate loss, so were not the focus of this study; and 2) after a parent died during the nesting cycle (*n* = 170), the remaining parent (widows and widowers) may re-pair with an individual who becomes a stepparent, or may not re-pair. Infanticide attacks at nests with stepparents were often carried out by stepparents. Nest abandonment occurred before, after, and in the absence of infanticide attacks. Five outcomes related to adoption and infanticide are distinguished by the context of attacks, the individuals attending the nests, and the fate of nests. Adopted nests were cared for by a widowed parent and a stepparent, and were not attacked or abandoned. Mixed nests had some nestlings killed by infanticide and others that were cared for by stepparents until fledging successfully. Infanticide is designated separately for nests attended by stepparents and those attended by others (widowed parents alone or intact pairs). These nests had eggs or nestlings that were attacked and either injured or killed. Infanticide by neglect occurred at nests that were not attacked, but where the offspring died as a result of nest abandonment. Sample sizes of nests are presented at each bifurcating step and percentages are presented when informative. Y = Yes, N = No. Nest fate refers to the number (and percent) for each couplet of successful nests fledging ≥1 offspring (S) and those that failed to fledge any offspring (F). An * indicates the number of nests that were abandoned before infanticide occurred and subsequently attacked. All but one of these 10 nests failed (the exception, with a stepfather, experienced infanticide after early-hatched chicks had fledged). Abandonment also occurred after an infanticide attack and some of these nests were successful if they had already fledged early-hatched chicks when abandoned. ^ signifies that the fates of two late-season nests were still being monitored when the field season ended.

Parrotlets wounded or killed nestlings and eggs at 256 (9.3%) of 2,742 nests monitored during the long-term study (Fig. 1). Infanticide attacks occurred in two distinct contexts. Offspring were attacked most frequently at nests with intact breeding pairs (*n* = 176 or 69%) where both parents were alive. Attacks occurred secondarily at nests where one mate had died (*n* = 80 or 31%), including 54 of 128 nests (42%) with widows and 16 of 42 nests (39%) with widowers. Infanticide by neglect in the absence of an attack was rare, occurring at eight nests where widowed parents re-paired with potential stepparents and then abandoned eggs or nestlings (Fig. 1).

Infanticide attacks resulted in the death of all offspring at 47% of nests (*n* = 121) (Fig. 1). However, at least one young fledged from 53% of attacked nests (*n* = 135), including 103 nests of intact pairs, 26 nests with widows, and six with widowers. Most of these nests with widowed parents (*n* = 21) were attacked shortly before or after several older siblings had fledged, yet contained younger chicks that were small enough to be killed due to the extreme hatching asynchrony of parrotlets (up to 17 d) (39, 41). The remaining nests (*n* = 11) contained some eggs or nestlings that were killed, and others that survived or were not attacked and received parental care until fledging. At 10 nests, harassment from other parrotlets led intact pairs (*n* = 2) and widowed males (*n* = 3) and females (*n* = 5) to abandon their nesting attempts before infanticide occurred, leading to death of eggs (*n* = 6 nests) or nestlings (*n* = 4 nests). Excluding nests abandoned before attacks, 718 nestlings were attacked and 603 nestlings and 251 eggs were killed from infanticide by parrotlets at active nests. None of the infanticide victims were partially consumed, and no biological parent was observed attacking offspring.

**Fig. 2.**
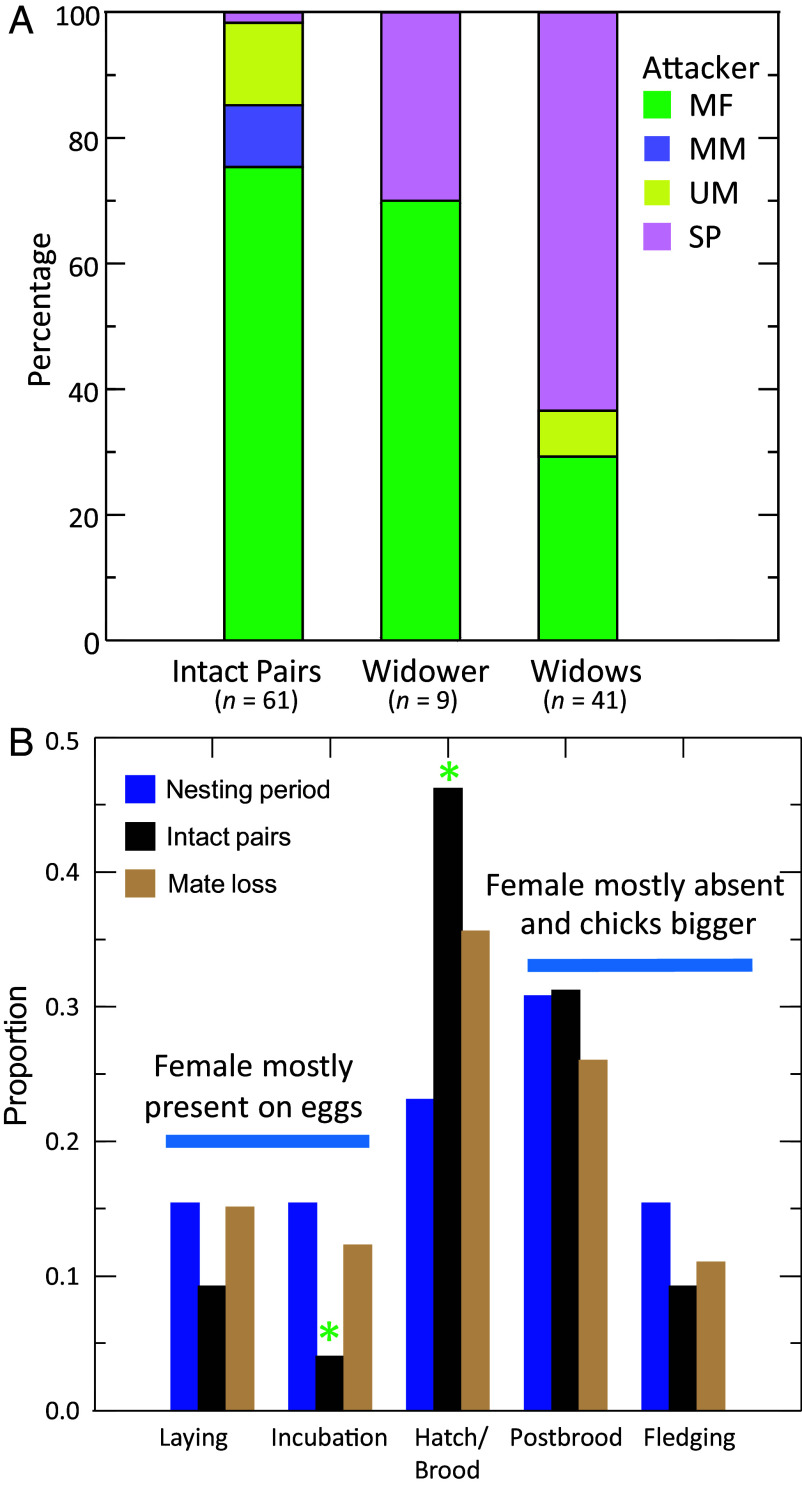
The social status of attackers and timing attacks during the nesting cycle in relation to the context of infanticide. (*A*) Social status of 111 attackers at parrotlet nests with intact pairs, widowers, and widows. Attackers: MF = male–female pair; MM = male–male pair; UM = unpaired male; and SP = stepparent. (*B*) Timing of infanticide during a typical 65-d parrotlet nesting cycle, from laying of the first egg through fledging of the last young. Females do all incubating and brooding, which ends by the time the largest chicks become homeothermic around 15 d posthatching. The fledging period is the time elapsed between fledging of the first and last-hatched young, which can be up to 2 wk due to asynchronous hatching. The blue bars represent the expected proportion of infanticide attacks based on the duration in days of each nesting period (laying = 10, incubation = 10, hatch/brood = 15, postbrood = 20, fledging = 10) for comparison with the proportion of events that took place at nests with intact pairs and with mate loss where one parent died. An * indicates a significant difference in proportions between nests with intact pairs versus the duration of the nesting period (likelihood ratio test, *P* < 0.05). None of the other comparisons between expected and observed attacks were significant.

Both sexes attacked and killed offspring, but males were 1.8 times as likely as females to be directly observed or suspected perpetrators (*n* = 77 and 42, respectively; *SI Appendix*, Table S1). Members of male–female pairs competing for nest sites accounted for three-quarters of the infanticide attacks at nests with intact breeding pairs (Fig. 2*A* and *SI Appendix*, Tables S2 and S3). One stepfather killed offspring at a nest with an intact pair where he displaced the male parent, a rare instance of divorce which occurs in only 1% of parrotlet pairs within a nesting season (37). At nests with widows, however, stepfathers were the predominant attackers, accounting for 63% of infanticide attacks, while attacks by male–female pairs occurred about half as often (Fig. 2*A*). Attackers were less frequently observed at nests with widowed males, where 67% of attacks were perpetrated by male–female pairs and 33% by stepmothers. Members of male–male pairs and unpaired males infrequently committed infanticide attacks (Fig. 2*A*). Nine individuals (six females and three males) participated in two infanticide events (i.e., “serial killers”), as either stepparents (two females) or members of male–female pairs (14 nests). They were directly observed (four times) or suspected (14 times) of killing offspring or eggs at nests of intact pairs (*n* = 8) or pairs that experienced mate loss (widows *n* = 2, widowers *n* = 4).

Infanticide attacks occurred throughout the nesting cycle but were more frequent at nests containing nestlings than at nests with eggs during laying and incubation (Fig. 2*B*), when they are protected by incubating females for >85% of the daylight hours (42). At nests with intact pairs, infanticide attacks occurred significantly less often during incubation and significantly more often during hatching and brooding (χ^2^ = 18.12, *df* = 4, *P* < 0.001) than expected by the duration of those portions of the nesting cycle (Fig. 2*B*). During hatching and brooding, females transition to foraging with males to provision chicks, which are small and easily killed by bites from adult parrotlets (*SI Appendix*, Figs. S1–S3). Infanticide attacks declined during the postbrooding and fledging periods when nestlings are harder for adult parrotlets to kill because they have grown larger and stronger, become more agile, and are protected by plumage and ossified skulls (Movies S1–S5). At nests with a mate loss, infanticide attacks occurred at the frequency expected by the duration of the stages of the nesting cycle (Fig. 2*B*) (χ^2^ = 2.85, *df* = 4, *P* = 0.584).

**Fig. 3.**
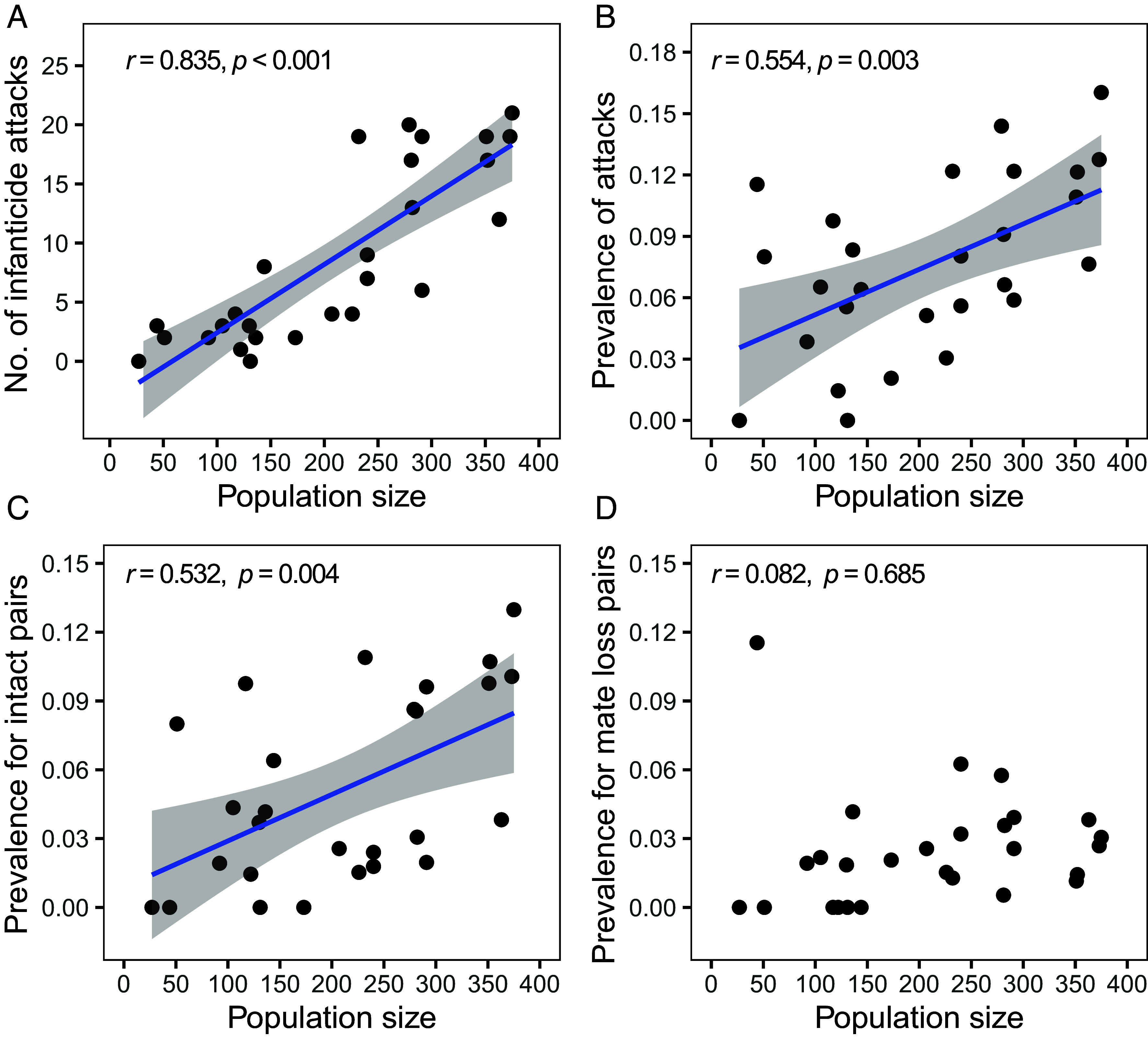
Population size effects on infanticide in the green-rumped parrotlet. Relationships between annual adult population size and the annual (*A*) total number of infanticide attacks, (*B*) prevalence of attacks (number of attacks/number of nesting attempts), (*C*) prevalence of attacks for intact pairs, and (*D*) prevalence of attacks for pairs with mate loss. Pearson correlation coefficient and associated slopes with 95% CI are depicted for significant relationships.

Infanticide was positively related to population size, but its influence differed by infanticide context. The number of attacks per year and prevalence of attacks (number per nesting attempt per year) were strongly and positively correlated with population size (Fig. 3 *A* and *B*). Likewise, prevalence of attacks on intact pairs showed an equally strong, positive correlation (Fig. 3*C*), indicating infanticide in this context was driven by competition for nest sites by other pairs (Fig. 2*A*). There was no relationship, however, between population size and prevalence of attacks on nests of widows and widowers (Fig. 3*D*), where attacks were carried out primarily by stepparents (Fig. 2*A*).

### Process and Outcome of Mate Replacement at Nests with Widowed Parents.

**Fig. 4.**
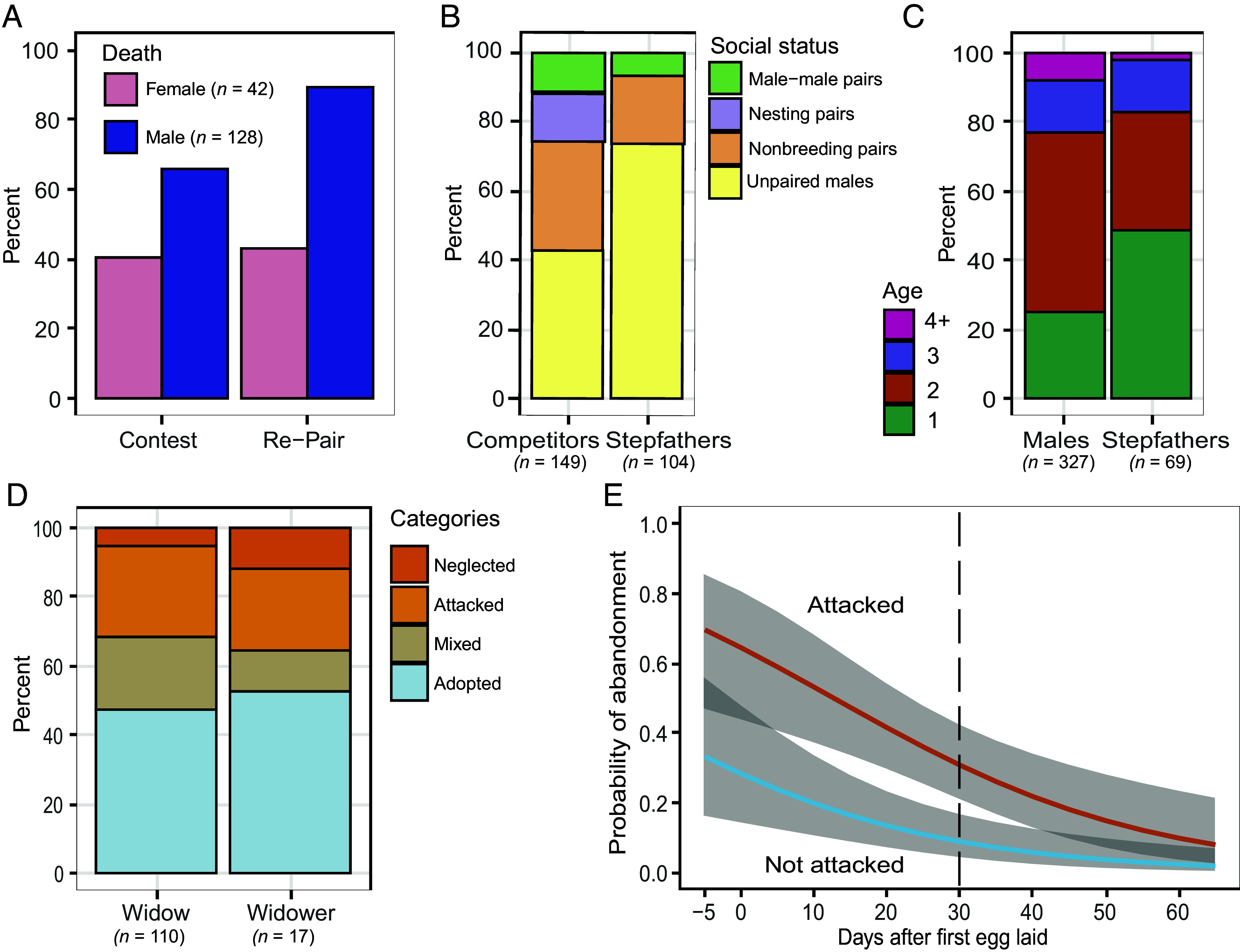
The process of mate replacement at nests after the death of a parrotlet parent, the social status and ages of competitors and eventual stepparents, and the outcomes of adoption and abandonment. (*A*) After the death of a male or female parent, the percent of nests that experienced a contest among competitors to become the stepparent, and the percent of widowed parents that re-paired with a stepparent. The complement to each bar is the percent of nests where the process did not occur. (*B*) Social status of competitors at 17 experimental nests where male parents were removed compared to the social status of stepfathers chosen by widows. (*C*) Age of first breeding of males in the population compared to the ages of stepfathers chosen by widows after their previous mates died. (*D*) Percent of nests of widows and widowers that experienced infanticidal attacks or neglect, were adopted (not attacked or neglected), and mixed (attacked but some offspring survived and received long-term care). (*E*) Probability of abandonment of a nest where one mate died that did and did not suffer an infanticide attack in relation to the number of days elapsed after the laying of the first egg of a clutch. The dotted line indicates the mean number of days after laying for nests where a mate died and 95% CIs are shaded. This figure depicts the best model from the model set in *SI Appendix*, Table S4.

Death of a male parent was accompanied by conspicuous contests 66% of the time (*n* = 128; Fig. 4*A*) that attracted up to 20 individuals (mean = 7.1 ± 0.3). Similar contests occurred at nests with widowers (40.5% of 42 nests) but significantly less often (χ^2^ = 8.86, *df* = 1, *P* = 0.003) and were attended by fewer participants (mean = 5.1 ± 0.6). At 17 nests where males were experimentally removed and we continuously observed the replacement process, small aggregations of parrotlets usually arrived within an hour of removal (*SI Appendix*, Fig. S4). Activity around the nest grew steadily, reaching a maximum number later in the day or early the next morning (mean = 15 daylight hours elapsed). Of the 149 banded individuals identified at intensively watched nests where males were experimentally removed, 43% were unpaired males, 32% were nonbreeding pairs that did not have nest sites, 14% were male–female pairs nesting nearby that were attracted to the commotion, and 11% were male–male pairs (Fig. 4*B*). Widows showed signs of pair bonding (e.g., allofeeding, allopreening, and joint defense of the nest site) on average 24 daylight hours after male removal (range: 1 to 56 h). Activity around the nest usually diminished by 30 daylight hours after mate removal (*SI Appendix*, Fig. S4).

Widowed females (*n* = 128) re-paired with a stepfather 90% of the time (Fig. 4*A*) but only 43% of the widowed males (*n* = 42) secured stepmothers, a significant difference (χ^2^ = 38.9, *df* = 1, *P* < 0.0001). Three-quarters of the replacements pairing with widows were previously unpaired males, 20% were males that dissociated from nonbreeding male–female pairs, and 6% were males that were previously members of male–male pairs (Fig. 4*B*). All females that paired with widowers and had been observed previously were members of nonbreeding pairs with different males (*n* = 11), which sometimes led to male–male conflict.

Males that became stepfathers tended to be young individuals (Fig. 4*C*). Nearly half of the replacement males of known age were 1-y olds, whereas the age of first breeding for male parrotlets was typically 2 y or older, a significant difference (χ^2^ = 24.58, *df* = 3, *P* < 0.0001). Moreover, 72% of replacement males banded as nestlings had no prior nesting experience. Becoming a stepfather was also a quick way to obtain a new mate for old males (5 to 9 y) who had previously nested but whose mates had recently died.

### Behavioral Ecology of Adoption.

Adoption was a common response of stepparents (Fig. 4*D*), whereas offspring were never adopted at nests with intact pairs that were attacked and evicted. We defined adoption broadly as nests attended by stepparents that were neither attacked nor neglected. No offspring mortality or injuries from infanticide occurred at 54% of 127 nests where stepparents joined widowed parents to care for offspring (Fig. 1: 58 of 110 nests with stepfathers, 11 of 17 nests with stepmothers). However, eight of these nests were neglected (abandoned) (Fig. 1: six with stepfathers; two with stepmothers). Thus, adoption occurred at 47% of nests with stepfathers (*n* = 52 of 110) and 53% of nests with stepmothers (*n* = 9 of 17) (Fig. 4*D*). The duration of care by stepparents was significantly longer (*t* = 3.41, df = 126, *P* < 0.001) at nests that were not attacked or neglected (17.4 ± 1.8 d) than those that were attacked (9.9 ± 1.3 d) ( *SI Appendix*, Fig. S5). However, 23 nests of widows with stepfathers (23%) and two of widowers with stepmothers (12%) exhibited a mixed response, where some nestlings were killed but others fledged successfully (Figs. 1 and 4*D*). Stepfathers at mixed nests provided long-term care after being attacked (mean = 17.3 ± 2.6 d), while mixed nests with stepmothers were close to fledging or failure and received short-term care (mean = 3.5 ± 0.5 d).

Stepparents of both sexes entered nests to feed offspring directly and stepfathers regurgitated seeds to their mates near the nest box, who then descended into the box to feed their nestlings. Adoptive stepparents frequently chased away other males and pairs that aggressively tried to take over the nest box or court the widowed parent, investing generalizable or shareable parental care toward eggs and young through nest defense. Parental care lasted significantly longer (*t* = 3.21, df = 70, *P* = 0.002) for stepfathers (22.6 ± 1.8 d) than for stepmothers (11.2 ± 2.3 d) (*SI Appendix*, Fig. S5). When female parents died at nests with eggs, abandonment and nest failure always ensued because stepmothers never incubated eggs and males do not perform incubation.

Terminating parental care through nest abandonment, rather than continuing to care for the offspring with (adoption) or without the help of a stepparent, was common. Nest abandonment by a widowed parent occurred at 22% of 170 nests (Fig. 1), and significantly more often (χ^2^ = 15.878, *df* = 1, *P* < 0.0001) at nests containing eggs (40%, *n* = 55) versus nestlings (13%, *n* = 115). We accounted for the latter effect by modeling the probability of abandonment in relation to the days elapsed between clutch initiation and abandonment (*SI Appendix*, Table S4). Whether a nest suffered an infanticide attack was as informative as the number of days elapsed, and the top model contained both factors (AICc weight = 0.736). Attacked nests were 3.5 times more likely to be abandoned (probability = 0.307) as nests that were not attacked (0.088) at the mean value of days elapsed (30 d), a significant difference (Fig. 4*E*). The sex of the widowed parent and whether it re-paired with a potential stepparent were uninformative (AICc weights = 0.001). Thus, abandonment was primarily a response to infanticide attacks, suggesting that aggression and neglect may often operate as sequential mechanisms of infanticide.

Stepparents that entered a nest box were both a threat to kill offspring and a potential provider of food and protection for them. Some stepfathers were aggressively prevented from entering nest boxes by widows; instead of entering nest boxes to feed offspring, these stepfathers sometimes regurgitated seeds to their widows near the nest, who then entered the box to feed offspring. At nests watched after mate loss (mean= 16 h, range: 2 to 60), 72% of adoptive stepfathers were not observed entering nest boxes (*n* = 32) compared to 40% of stepfathers at nests that were attacked (*n* = 15). This difference was significant (χ^2^ = 4.39, *df* = 1, *P* = 0.036), suggesting the occurrence of infanticide may be influenced by the degree that widows inhibited attacks by stepfathers.

There were no other measured differences found between adoptive and infanticidal stepfathers. Ages of adoptive (2.33 ± 1.87 y, *n* = 36) and infanticidal (2.06 ± 1.53 y, *n* = 31) stepfathers (*SI Appendix*, Fig. S6) did not differ (Kruskal–Wallis χ^2^ = 0.08, *df* = 1, *P* = 0.783). Similarly, the percent of replacement males with no prior nesting experience did not differ (Fisher’s exact test, *P* = 0.794) between adoptive (71.1%, *n* = 38) and infanticidal stepfathers (74.2%, *n* = 31). Moreover, the probability of adoption by stepfathers was unrelated to the date of predecessor mortality within the nesting season (logistic regression, β = 0.246, *n* = 101, *P* = 0.202) or to population size (logistic regression, β = −0.001, *n* = 99, *P* = 0.829).

### Fitness Consequences of Infanticide and Adoption.

**Fig. 5.**
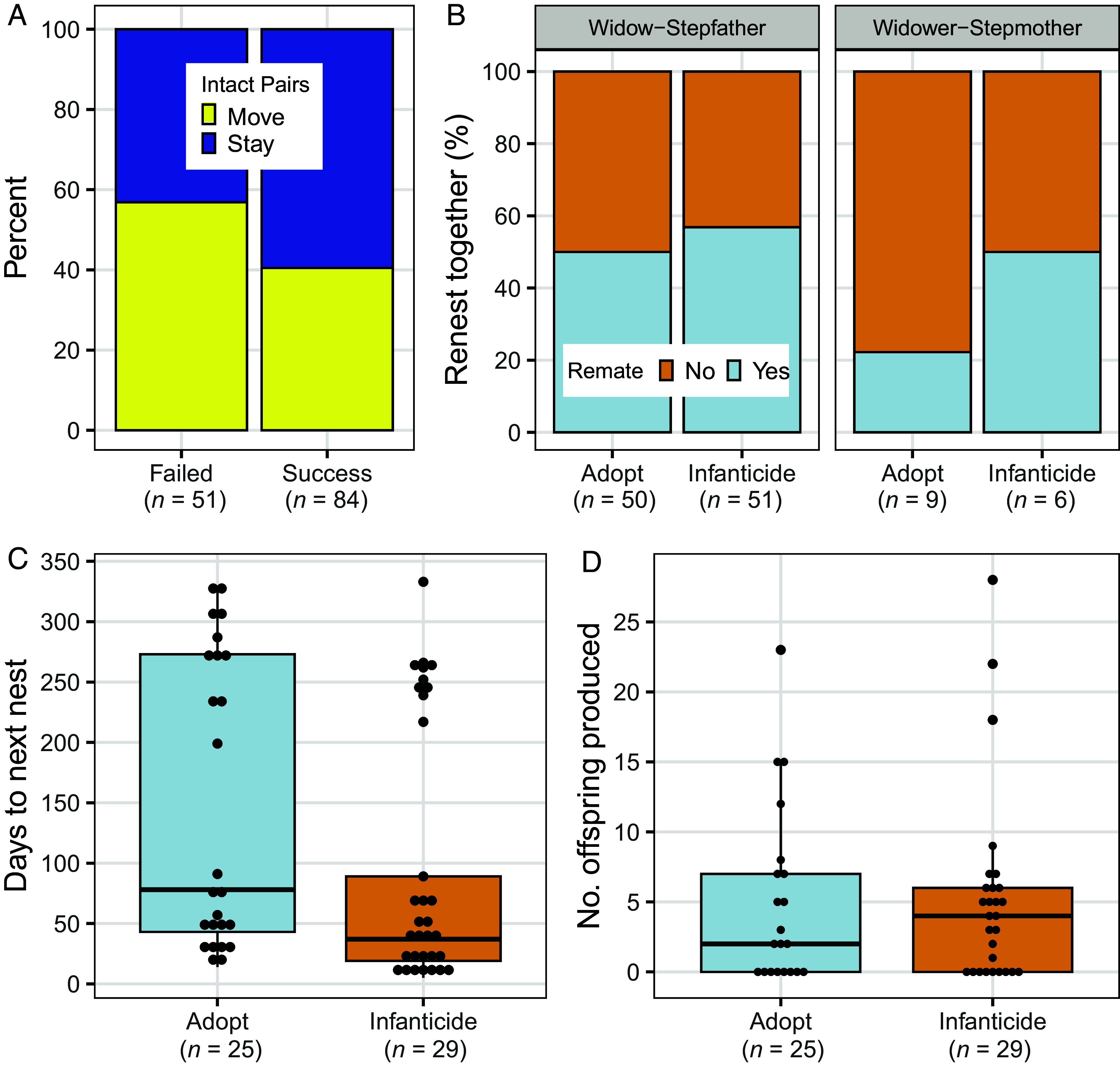
Fitness consequences of infanticide and adoption. (*A*) The probability of intact pairs being evicted and moving to a new nest site after an infanticide attack if their nest failed or succeeded. (*B*) The probability of a stepparent nesting in the future with the widowed parent. (*C*) The time to laying of the first egg at the next nesting attempt. (*D*) The number of young produced by a stepfather with a widowed female in all future nesting attempts.

Infanticide attacks at nests with intact pairs offered a way for pairs without active nests to evict owners and obtain a nest site. Of 171 intact pairs that experienced infanticide attacks on their eggs or nestlings, 42% renested in the same site, 37% moved to a new cavity and 21% never nested again. Furthermore, intact pairs that experienced infanticide were significantly more likely (χ^2^ = 3.424, *df* = 1, *P* = 0.032 one-tailed test) to move to a different site if their nest failed than if it successfully fledged young (Fig. 5*A*). We documented 29 nest boxes of intact pairs suffering infanticide attacks that were usurped and subsequently served as nests in the same breeding season for individuals observed or suspected of committing infanticide (*n* = 12), or for other male–female pairs previously lacking nest sites (*n* = 17). Two-thirds of usurping pairs nested only once in the box, but the remainder nested two to five times over 2 to 4 y, producing up to 26 fledglings (mean = 4.2 fledglings per box). Members of intact pairs that experienced infanticide attacks did not nest with observed or suspected attackers except in one instance.

There were clear fitness benefits to becoming a stepfather, but few differences in fitness between adoptive and infanticidal stepfathers. The greatest benefit for a stepfather was acquiring a potential mate and sometimes a nest site: 53% of stepfathers (*n* = 101) nested in the future with widows (Fig. 5*B*) and 69.8% of these stepfather-widow pairs subsequently nested in the widow’s box (*n* = 54 pairs). However, adoptive stepfathers had a similar chance (χ^2^ = 0.478 *df* = 1, *P* = 0.489) of nesting in the future (Fig. 5*B*) with their widow (50%) as did stepfathers that committed infanticide attacks (57%). Moreover, the proportion of stepfathers that retained the nest box did not differ (χ^2^ = 0.006 *df* = 1, *P* = 0.939) between nests that were attacked (69.0%, *n* = 29) and were not attacked (68.0%, *n* = 25). Although infanticidal stepfathers nested with widows significantly sooner (Kruskal–Wallis χ^2^ = 5.94, *df* = 1, *P* = 0.015) than adoptive stepfathers (medians: 37 versus 78 d later; Fig. 5*C*), the number of future offspring produced with widows (Fig. 5*D*) did not differ significantly between them (Kruskal–Wallis χ^2^ = 0.031, *df* = 1, *P* = 0.861). The sample size for stepmother-widower pairs (*n* = 15) was too small for meaningful comparisons of fitness measures.

There was no evidence that stepparents benefitted strongly by kin selection. Based on pedigrees of banded individuals, none of the stepfathers (*n* = 66) or stepmothers (*n* = 8) were first-degree relatives of their widows or widowers, respectively, or the original male/female parent that had died.

## Discussion

Two surprising findings emerged from our long-term study of infanticide and adoption in the green-rumped parrotlet. First, we expected infanticide to occur primarily at nests where a parent died, or was displaced by an unmated male (9). This form of infanticide by stepparents did occur, but the dominant context in parrotlets was attacks on offspring of intact nesting pairs, usually by nonbreeding male–female pairs (Figs. 1 and 2*A*). Infanticide through eviction occurred twice as often as infanticide associated with stepparents at nests where one parent had died. Eviction-driven infanticide yielded fitness advantages to the killers by inducing some pairs to relinquish their nest sites. This allowed evicting pairs without a nest cavity to get one (Fig. 4*A*), and facilitated the movement of evicting pairs with unproductive nest sites to more productive cavities they sought to obtain through infanticide (40). As predicted, the prevalence of eviction-driven infanticide was positively related to population size (Fig. 3*C*), confirming strong density-dependent effects on infanticide (31). Since the divorce rate of intact pairs is only 1 to 2% within and between years (37), eviction-driven infanticide in parrotlets was caused primarily by resource competition for nest sites.

Second, adoption was a common response of stepparents at nests where a parent had recently died, occurring about as often as infanticide (Fig. 4*D*). Among vertebrates, adoption occurs sporadically across a diversity of fishes (43), birds (6, 10, 44), and mammals (45–47). As expected when adult sex ratios are skewed toward males (48), parrotlet widows nearly always re-paired quickly (Figs. 1 and 4), often with unmated males that were young and lacked previous nesting experience. In comparison, less than half of the widowers recruited stepmothers, who were always nonbreeding females paired with a different male.

Infanticide and adoption by parrotlet stepparents were not uniform patterns of behavior (18, 49) or binary conditions but represent a continuum of facultative behaviors. Infanticide by stepparents typically took the form of aggressive attacks. If all offspring had not been killed, however, aggression could be followed by neglect (abandonment), which was far more common after, than independent of, an infanticide attack (Fig. 4*E*). Thus, aggression and neglect often acted in concert as sequential rather than separate mechanisms of infanticide in parrotlets. Moreover, infanticidal and adoptive behaviors of stepparents occurred in the same nest. A mixed response was exhibited by 23% of stepfathers with widows (Figs. 1 and 4*D*), where some nestlings were killed but others received long-term care until fledging. Finally, some widows successfully mitigated aggression by stepfathers by preventing them from entering nest boxes, and their offspring were less likely to be attacked than those that did not prevent stepfathers from entering. Infanticide prevention strategies are well known in mammals (11, 20, 21), but have not been previously described in birds.

Becoming a stepparent—either adoptive or infanticidal—offered clear advantages to parrotlets of both sexes. By remating with widows, stepfathers attained an earlier age of first breeding than did their competitors (Fig. 4*C*) and often inherited the widow’s nest box, securing a key resource. For stepmothers, adoption provided an opportunity to secure a widowed male with a nest site. Thus, parrotlet stepparents exhibited both sexually selected adoption, where stepparenting acts as a form of mating effort (6, 7, 10), and sexually selected infanticide typically observed in birds (9, 50).

While there were clear fitness benefits for becoming a stepparent, we found no difference in fitness (Fig. 5) between infanticidal and adoptive stepfathers (the sample of stepmothers was too small to test). Veiga (9, 14) noted few studies of birds demonstrate the advantage of rapid remating typically associated with male take-overs and infanticide in mammals (1, 12, 13). Parrotlet stepfathers at nests that were attacked did renest sooner than adopters (Fig. 5), but the saved time did not translate into increased offspring produced over the lifetime of the new pair, which may extend for multiple years. Thus, adoption and infanticide were strategies with equivalent fitness outcomes for parrotlet stepparents.

In conclusion, we have shown that infanticide within a single species and mating system occurs in multiple contexts (eviction-driven and sexual selection). Moreover, tolerance of and active investment in unrelated offspring by parrotlet stepparents were not maladaptive responses invoked by the stimulus of normal parental care. Instead, they represent adaptive behavior in the form of mating effort that often leads to future reproduction with the widowed parent (i.e., sexually selected adoption). Both infanticide and adoption in parrotlets were related to limited breeding opportunities driven by a relative shortage of cavities (i.e., resource competition leading to evictions) and mates (sexual selection). These conditions are exacerbated in parrotlets by their long-term, monogamous pair bonds, a strongly male-biased sex ratio that varies little across years, and protracted development required by extremely altricial offspring in a large-brained species (36, 37, 51). While monogamy in primates is thought to be a response to the risk of infanticide (12), long-term monogamy in parrotlets contributes to both infanticide and adoption.

## Materials and Methods

### Study Area, Study Species, and Monitoring Methods.

We studied green-rumped parrotlets from 1988 to 2015 at Hato Masaguaral (8°34′ N, 67°35′ W), a working cattle ranch 45 km south of Calabozo in the state of Guárico, Venezuela (32, 37). The habitat is flat, brushy *llanos* or savanna (52), and large areas flood during the rainy season (May to November). Fieldwork was conducted daily throughout the nesting season from early June through early December.

Parrotlets can initiate multiple nesting attempts per year and lay clutches of 4 to 12 eggs (median = 7) over 6 to 17 d (39, 41). Females do all incubation, beginning on the first egg (42), which leads to extreme hatching asynchrony and frequent mortality of last- and penultimately hatched young in large broods (39). Males guard their mates when they leave the nest box, provide nearly all of the female’s food during incubation, and participate with females in feeding nestlings (32, 53). Slow development of embryos (54) and growth of chicks (55) results in eggs requiring 17 to 24 d of incubation (42) and chicks fledging at 26 to 41 d after hatching (39). Thus, a successful nesting attempt requires on average 65 d (47 to 78 d) from laying of the first egg until fledging of the last young (54), during which time the eggs and young are susceptible to infanticide. Nest sites are in short supply and vary tremendously in productivity; nests in boxes that produce more offspring suffer more infanticide attacks (40).

Parrotlet nests range from semicolonial associations, with active nests only a few meters apart in the same tree, to isolated nests. In 1988 and 1989, 106 identical nest boxes, made of 1-m deep polyvinyl chloride tubes with hardware cloth interiors (56), were installed no closer than 10 to 20 m apart along fence lines in a 4 km^2^ study area (37, 40). The minimum distance between boxes was based on observations of simultaneously active nests in natural cavities. Nest boxes were rapidly accepted and replaced natural cavities as the most frequently used sites (56, 57). We monitored an additional 20 nest boxes in some years in a third population 2.5 km from the others. Across the 27-y period of this study (1989 to 2015), a team of two to five field researchers annually monitored the fate of all nesting attempts in boxes (total: 2,742; mean = 100.2 ± 10.5 per year, range: 11 to 187) following methods described in the next section.

We captured 1,512 breeding and nonbreeding adults with mist nets. They were banded with individually identifiable color bands and a numbered metal band, as were all nestlings (*n* = 7,346) (37). Sex of nestlings and adults was determined by differences in plumage color (58), and the number of offspring fledged was determined 1 to 3 d prior to fledging. Repeated resightings (>45,000) of uniquely color-banded individuals from daily surveys conducted throughout the 6 to 7 mo nesting season confirmed the identities of parents attending nests and nonbreeders. Adults are highly philopatric and the resighting rate of breeders is near unity (34, 35). Thus, breeding adults not resighted likely died. The total number of after-hatch-year individuals observed annually based on identities of color-banded birds (mean = 209.4 ± 20.2, range: 27 to 373) was used as an estimate of adult population size.

### Evidence of Infanticide and Mate Loss.

Nest boxes were visited daily every 1 to 3 d throughout the entire nesting season to determine nest contents and the identities of adults in the vicinity. Data collected from these checks, combined with 2,140 h of nest watches and video recording in nests, provide strong evidence for the occurrence of infanticide and mate loss as well as adoption and nest abandonment (see next section). Thus, with the exception of 17 experimental nests described below, infanticide was primarily detected after an attack occurred by finding injured or dead nestlings in the box and was secondarily detected when we opportunistically encountered an event in process.

Parrotlets are unable to remove items from their meter-deep nest box because they use their bill and legs to ascend the nest cavity. Thus, nestlings or eggs killed by infanticide, or that died from other causes like starvation, remained in the nest box where we examined them during routine nest checks. In contrast, predation resulted in an obvious reduction in the number of eggs or young in the nest between nest checks. Predation on nestlings and eggs was easy to distinguish from infanticide attacks by the patterns of mortality and injuries (40, 57). Predators usually consumed all nestlings or eggs, leaving no trace of the missing offspring in the nest box. Infrequently a predator consumed all but one or two eggs or young, or left behind a smashed egg or pieces of consumed chicks.

Nestlings attacked by parrotlets usually exhibited bruising to the head, but also to the neck, back, wings, and/or legs, with visible internal hemorrhaging and external lacerations (*SI Appendix*, Figs. S1–S3). Nestlings killed by parrotlets never showed signs of being consumed. Eggs attacked by parrotlets were often punctured rather than smashed, and frequently had triangular bite marks matching the size and shape of a parrotlet bill (33, 57). Nest checks also allowed us to determine visually whether nestlings had been fed by parents based on their crop contents (39).

We found no evidence that nestling injuries were inflicted by parrotlet siblings. We never detected aggressive interactions between nestlings during tens of thousands of nest checks and extensive handling of nestlings. Moreover, we never observed nestlings to vigorously bite or attack each other during ~2,000 h of video observations inside boxes at 51 nests as part of our studies of vocal development (59–62). Camcorders recorded continuously, beginning between 0700 to 0900 h and lasting 1 to 3 h. Filming was conducted daily after the female parent ceased incubation and continued until the last nestling fledged from the nest or the nest failed. Data were stored as Advanced Video Coding High Definition files on multiple external hard drives. Adobe Audition (v13.0.3.60, 2020, Adobe Inc.) was used to review video. We did, however, record two instances of infanticide attacks (e.g., Movies S1–S3).

Saffron finches (*Sicalis flaveola*), straight-billed woodcreepers (*Dendroplex picus*), and brown-crested flycatchers (*Myiarchus tyrannulus*) occasionally prospected the nest boxes and infrequently initiated nests in unoccupied boxes. Parrotlets were dominant over all three competitors, displacing them on or near nest boxes and chasing them away. We never observed these species entering unguarded nest boxes and attacking parrotlet nestlings or eggs. On the other hand, our video monitoring of woodcreeper nests detected several instances of parrotlets attacking woodcreeper adults or offspring in nest boxes.

The obvious commotion at nest boxes from contests among parrotlets alerted us to the death of a parent (which was confirmed by resighting efforts during the current and subsequent years) and attempts to evict breeding pairs from nests (Movie S4). In these cases, up to 20 parrotlets were observed competing at the nest box through vigorous vocal and visual displays, fighting, and courtship or harassment of the widowed parent or nesting pair. These nests were often watched for periods of 1 to 3 h to identify the individuals present, note their social status, and record their behaviors, and thereafter were revisited regularly to determine the fate of the nest and the identity of the attending birds. Social status was classified as actively nesting male–female pairs, nonbreeding male–female pairs, unpaired males, and male–male pairs (37). Male–male pairs comprise 10% of nonbreeding males, are typically young (<1 y of age), are rarely composed of siblings, and affiliate together from 2 to 12 mo (37).

Watches at nests led to direct evidence and documentation of the killing or attacking eggs and nestlings by 24 parrotlets (14 males, 10 females) at 18 nests. In those instances, we observed nonbreeding parrotlets or stepparents entering and/or emerging from nest boxes that contained parrotlet young or eggs. Sometimes blood was observed on the bill of the departing bird. Shortly after the intruding parrotlet departed, checks of the nest contents by observers documented the killing or wounding of nestlings or eggs. At another 93 nests, we identified suspected killers as those individuals that were observed repeatedly to enter or try to enter nest cavities, perch on or near the box, and/or extensively harass the nesting pair or widowed parent. The social status of observed and suspected attackers was similar and did not differ significantly (*SI Appendix*, Table S2), so we combined observed and suspected for subsequent analyses (*SI Appendix*, Table S3 and Fig. 2*A*).

### Criteria and Evidence for Adoption and Abandonment.

We classified stepparents as adopting unrelated offspring if no offspring mortality or injuries occurred from infanticide attacks, and stepparents exhibited forms of parental care, ranging from feeding of offspring to tolerance (10). Our definition follows Avital et al. (49) in contending “*adoption is not a uniform pattern of behavior”* and includes what Daly and Perry (7) called the “*middle ground*” between active parenting and infanticide (i.e., tolerance). Part of the challenge in defining adoption is the difficulty of distinguishing parental care from mating effort from resource defense, since the same behavior (e.g., nest defense) can serve all three purposes. We included multiple forms of caring behavior in our definition of adoption, instead of trying to partition parental investment from mating effort and resource defense.

In the green-rumped parrotlet, parental care and tolerance take multiple forms. Stepparents feed offspring directly and stepfathers regurgitate seeds to their mates, who then enter the nest box to feed their offspring. The latter feedings seem unlikely to function as a mating effort for stepfathers since the laying of a new clutch occurred on average 2 mo later. Stepfathers also invest generalizable or shareable parental care (time and energy) toward eggs and young through nest site defense and defense of the female by guarding her throughout the nesting cycle from harassment by unmated males. All of these forms of investment are important to the ultimate success of a nesting attempt for parrotlets.

Daily nest checks and periodic nest watches (765 h at 95 nests) and video monitoring (325 h at four nests) allowed us to observe whether stepparents exhibited adoption behaviors and to determine whether parents abandoned the remaining eggs and/or nestlings before or after an infanticide or mate loss event. Nest watches and subsequent daily visits to the box determined whether stepparents entered the nest box or were prevented from doing so by the surviving parent, fed their new mates outside of the box, and participated with the widowed parent in defending the box from other parrotlets. Nests were classified as abandoned when multiple visits over several days determined that nestlings had not been fed (i.e., had empty crops and symptoms of dehydration or malnourishment), eggs were not incubated (i.e., were cold, unattended, or buried in the bottom of the box), parents and stepparents were not nearby, and other parrotlets were prospecting at the box or entering it.

### Experimental Removal of Male Parents.

To observe the entire process of replacement of a deceased parent, 17 males were experimentally removed from active nests in 2002 (5), 2004 (6), and 2005 (6). Males were released 43 km from the study site within 2 h after removal; none were resighted during the same year, but two males returned by the following nesting season. Three males were removed during laying, five during hatching, and nine during brooding or shortly after it had ended. Experiments were spread throughout the first 3 mo of the nesting season and never later than 3 mo before the last breeding attempt each year to allow enough time for renesting. Nests were geographically dispersed throughout the study area to limit the social disruption caused by mate loss and to avoid overlap of potential individuals competing to become stepfathers. As a result, an average of 87.9% of all individuals identified at experimental nests were unique to each mate-loss event.

We conducted nearly continuous daylight observations (*n* = 1,050 h) beginning with removal of a parent and lasting 3 d postremoval (or until the nest failed). All individuals within 5 to 10 m of the nest box were identified when possible, and their social status and behavior toward the widow and the nest box noted. A count of individuals within a 20 m radius of the nest box was taken every 15 min.

### Data Analyses.

Data were managed with Microsoft Access 2016 and analyzed with Program R version 4.1.3 (63) and GraphPad (www.graphpad.com/). We tested for dependence of infanticide by correlating annual adult population size with the annual number or prevalence of infanticide attacks. Prevalence is the number of attacks per year divided by the annual number of nesting attempts, which yields the annual probability a nest was attacked. Prevalence was calculated annually for all attacks, intact pairs (number of attacks on intact pairs/number of nesting attempts), and widowed pairs (number of attacks on mate loss pairs/number of nesting attempts). Frequency differences were tested using chi-squared and Fisher’s exact tests. Differences between adoptive and infanticidal stepparents were compared using Kruskal–Wallis tests for continuous variables. Logistic regression tested for the occurrence of adoption and infanticide within the nesting season, after dates were centered and standardized within years. It was also used with Akaike’s information criterion corrected for small sample size (AICc) to model the probability of abandonment at nests of widowed parents in relation to days after laying of the first egg, its quadratic form, sex of the widowed parent, whether it re-paired with a potential stepparent, and whether an infanticide attack occurred. Two-tailed tests were employed except where noted. Statistical significance was set at *P* ≤ 0.05. Means are presented with SE.

## Supplementary Material

Appendix 01 (PDF)

Movie S1. Stepfather green-rumped parrotlet (#7920) attacks a brood of seven nestlings as the widow (A201) watches in foreground (Nest# 12B104A). All nestlings were repeatedly attacked by 7920, a 2-year-old who had yet to breed, over the following weeks, resulting in fatalities of the youngest nestling (24 October 2012). Six larger nestlings fledged successfully. The new pair did not go on to nest together.

Movie S2. A nonbreeding male green-rumped parrotlet (#8557) attacks nestlings of an intact breeding pair (Male X645, Female A216) (23 July 2019). Near the end of the movie, blood from a nestling can be seen flowing from a wound on the head to the bill of the adult. This one-year-old male (#8557) killed three young nestlings, but four nestlings eventually fledged successfully from this nest (Nest# 19B104A). Seeds are visible in the crops of several nestlings, so the parents had been providing food. Parents X645 and A216 had nested together the previous year, and they went on to nest again in 2019 after this nest attempt was completed. Black markings on the nestlings’ heads were used for individual identification during a study of vocal development.

Movie S3. Continuing on in time from Movie S3, the nonbreeding Male #8557 attacks the bill and head of another small nestling in Nest# 19B104A.

Movie S4. Contest among multiple male green-rumped parrotlets harassing an intact breeding pair (15 July 2019) at Nest# 19B31A. The male parent can be seen at the entrance of the nest box and his mate at the top. A fight ensues.

Movie S5. Normal parental feeding behavior exhibited by an intact male-female parrotlet pair passing food by regurgitating seeds into the mouths of their nestlings at Nest# 19B105B (26 Oct 2019).

## Data Availability

Data have been deposited in Dryad (DOI: 10.5061/dryad.h70rxwds4) (64).
