# Supplementary material for: Eviction-driven infanticide and sexually selected adoption and infanticide in a neotropical parrot
Source: Proc Natl Acad Sci U S A. 2024 May 6;121(20):e2317305121. doi: 10.1073/pnas.2317305121 (PMC11098109; doi:10.1073/pnas.2317305121)
Supplement: Supplementary file 1 — Appendix 01 (PDF) [file pnas.2317305121.sapp.pdf]

PNAS

Supporting Information for

Eviction-driven infanticide and sexually-selected adoption and infanticide in a Neotropical parrot.

Authors: Steven R. Beissinger and Karl S. Berg

Corresponding author: Steven R. Beissinger
Email: beis@berkeley.edu

This PDF file includes:

Figures S1 to S6
Table S1 to S4
Legends for Movies S1 to S5

Other supporting materials for this manuscript include the following:

Movies S1 to S5

Supplementary Figures:

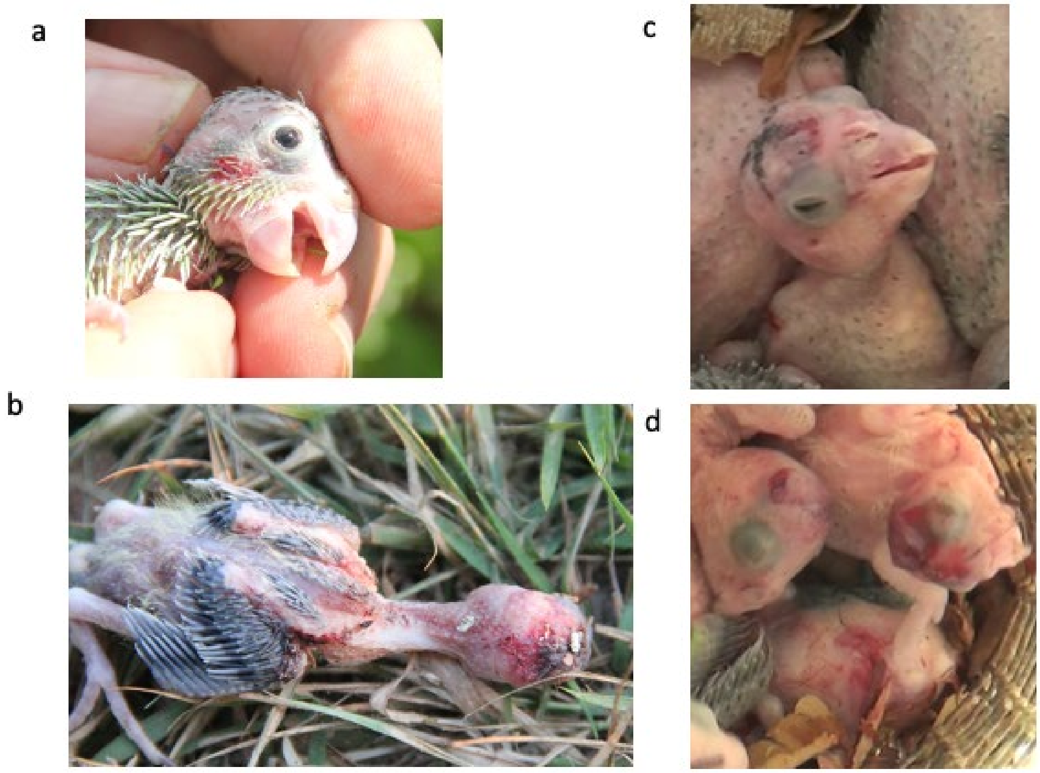

Supplementary Figure S1. Infanticide attacks on green-rumped parrotlet (*Forpus passerinus*) nestlings show bruising that resulted in internal hemorrhaging and external lacerations from bites to the head and body (note triangular bill-shaped wounds). **(a)** Nestling recently attacked by stepfather showing a fresh wound to the head. **(b)** Deceased sibling of individual in (a) after succumbing to attacks by stepfather, showing numerous wounds (28 Oct. 2012). (**c and d**) Nestlings recently attacked by a nonbreeding male during a brief absence by parents. Two died moments later (23 July 2019).

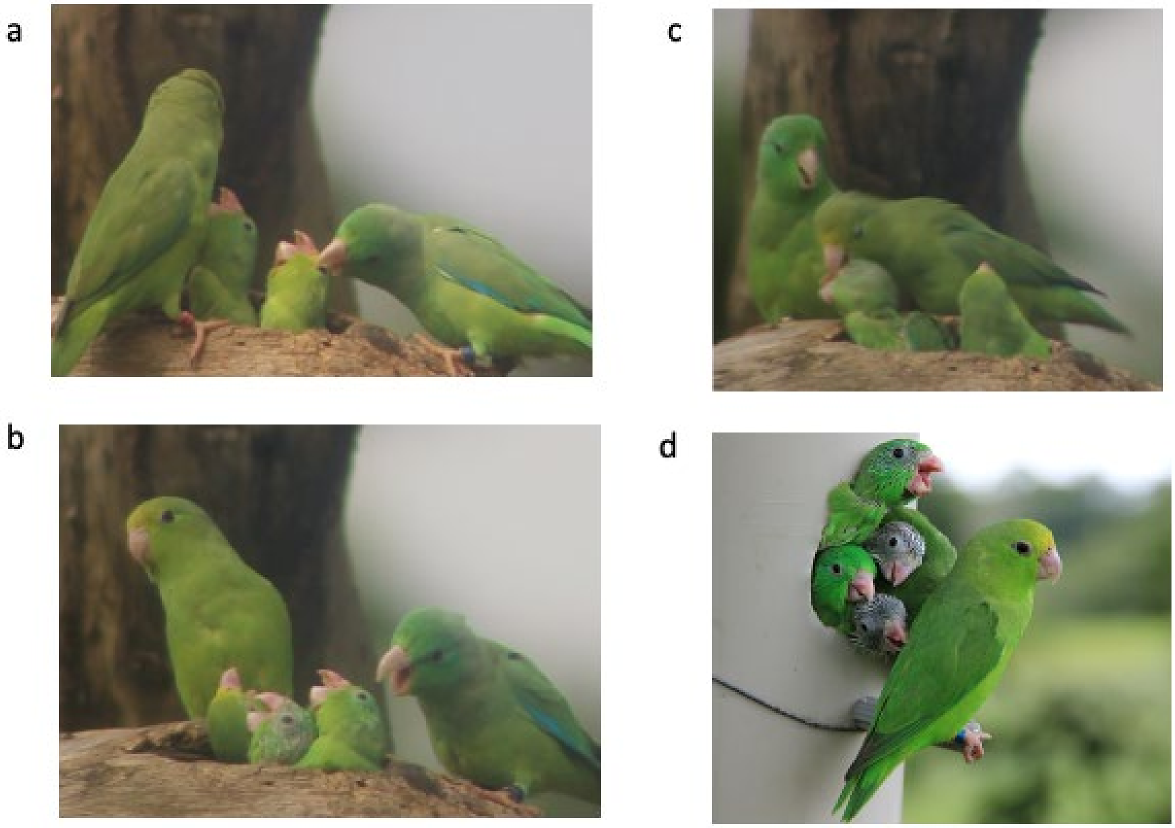

Supplementary Figure S2. **I**nfanticide and adoption in green-rumped parrotlets. (a-c) Repeated attacks by a nonbreeding pair on the nestlings in a natural cavity after both of their parents had disappeared and apparently died (23 Oct. 2012). (**d**) A recent widow shown with her brood, which she successfully raised with the help of a stepfather (27 July 2013).

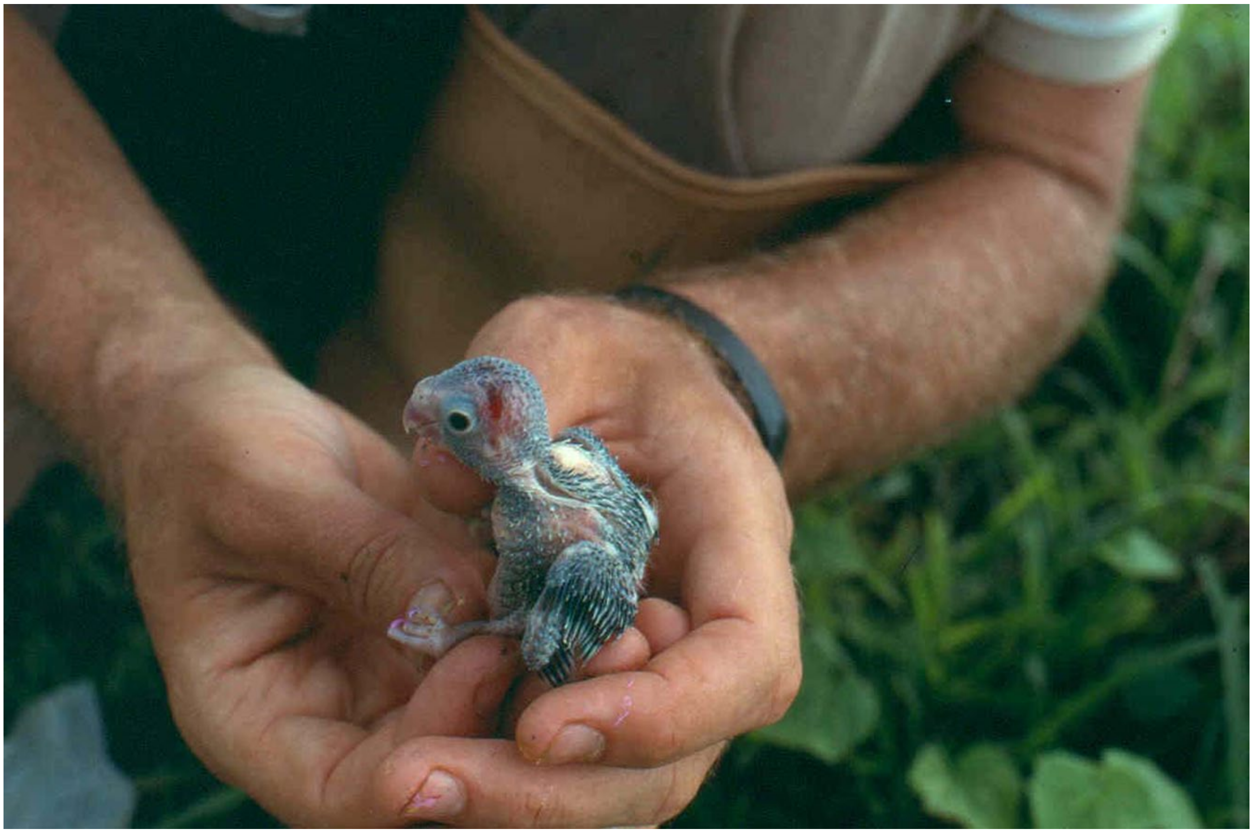

Supplementary Figure S3. A two week old parrotlet chick with head wounds suffered from an infanticide attack. The wounds have begun to heal and the chick eventually fledged.

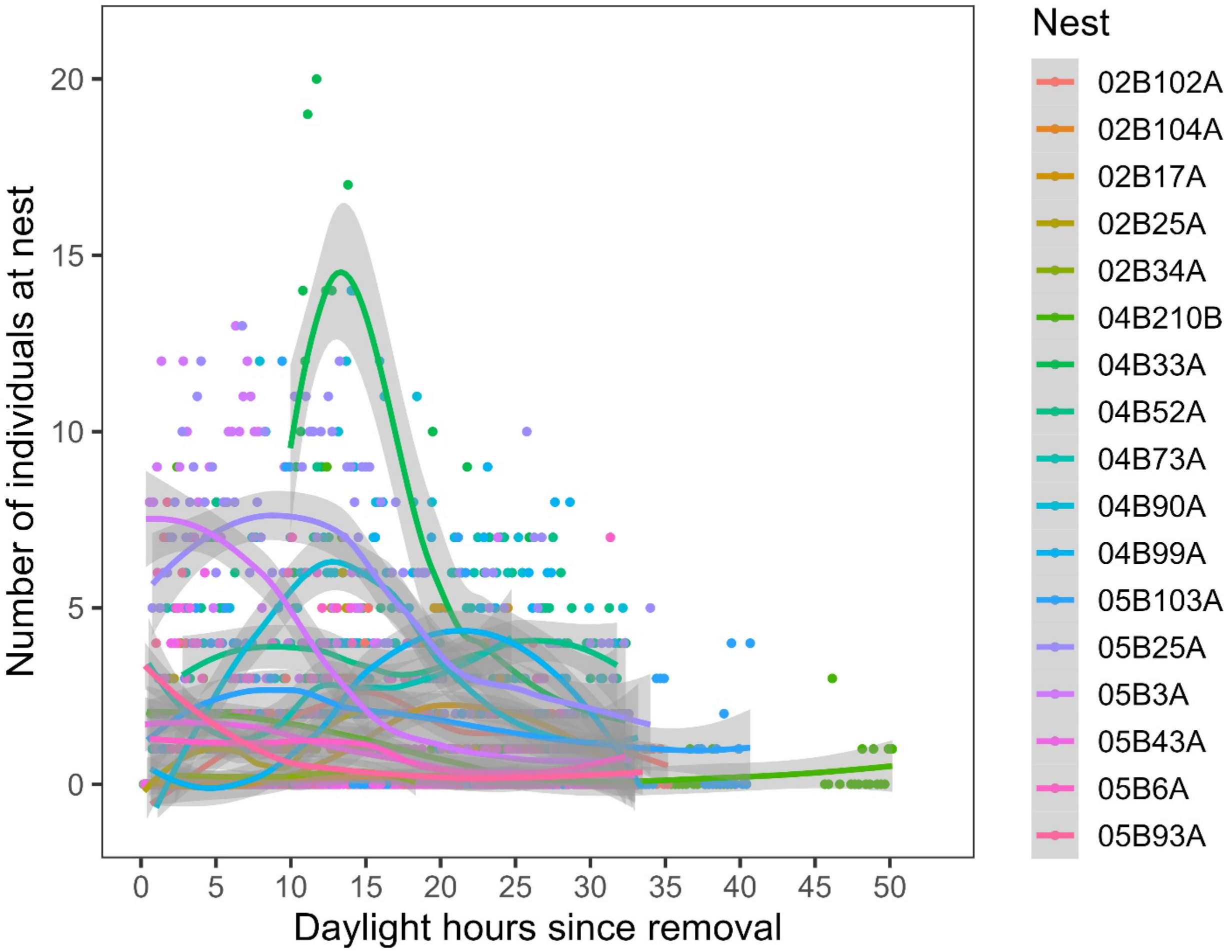

Supplemental Figure S4. The number of individuals attending contests at experimental nests where male parents were removed as a function of daylight time since removal. Data are from 1629 counts of individuals in the immediate vicinity of nests (20m radius) conducted every 15 minutes for the 3 days subsequent to the experimental removal of 17 breeding males. A loess smoother is shown for each nest with a 90% confidence interval.

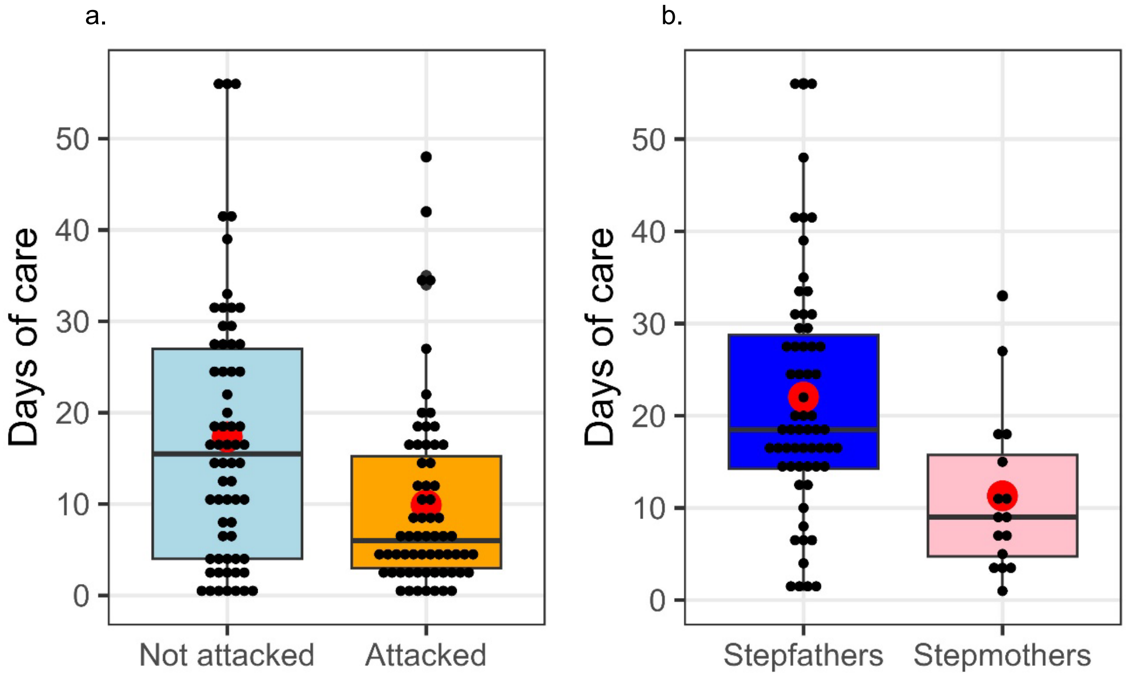

Supplementary Figure S5. The duration of parental care (number of days) by: (a) stepparents at nests that were not attacked or neglected compared to those that were attacked or neglected (*n* = 128) and (b) at 72 nests that were adopted by stepfathers and stepmothers. Dots indicate each nest. The median is shown by the solid line and the mean by the red circle. The duration of care by stepparents was significantly longer (t = 3.41, df = 126, p < 0.001) at nests that were not attacked or neglected. Stepfathers provided care significantly longer than stepmothers (t = 3.21, df = 70, p = 0.002

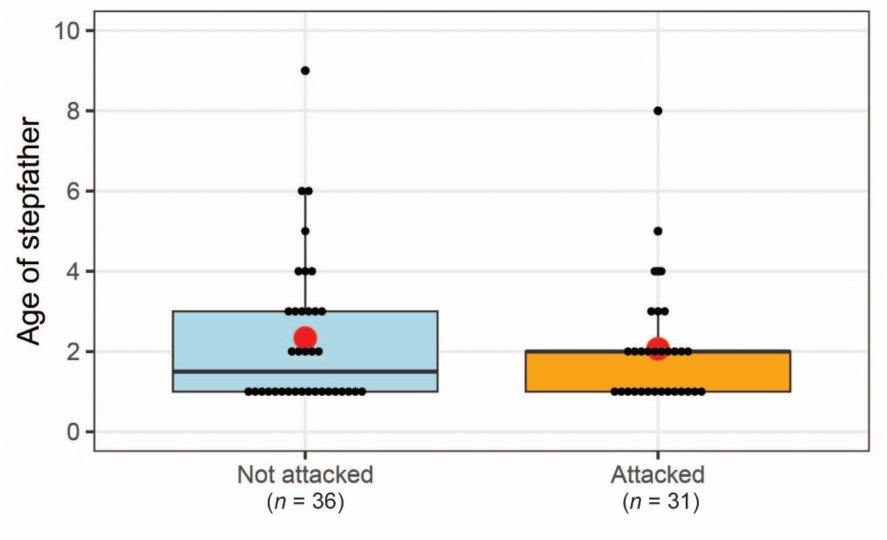

Supplementary Figure S6. Ages of green-rumped parrotlet stepfathers banded as nestlings at nests where offspring were adopted or attacked**.** Dots indicate age of stepfather at each nest. The median is shown by the solid line and the mean by the red circle. Ages of adoptive (2.33 $\pm$ 1.87 years, n = 38) and infanticidal (2.06 $\pm$ 1.53 years, n = 31) stepfathers did not differ (Kruskal-Wallis $\chi^2 = 0.08$, $df = 1$, $p = 0.783$).

Supplementary Tables

Table S1. Sex of 119 individuals directly observed (Seen) committing infanticide attacks and those suspected of committing attacks at 111 green-rumped Parrotlet nests. Observed attackers were individuals that were seen entering and/or emerging from nest boxes that contained parrotlet young or eggs. After the birds departed, checks of the nest contents by observers documented the killing or wounding of nestlings or eggs. Suspected individuals were frequently observed attempting to enter nest boxes, perching on or near the box, and/or harassing and fighting with the intact nesting pair or widowed parent after a mate loss. We lacked sufficient evidence to determine the identities of attackers at 135 additional nests with eggs or chicks that were killed or wounded and exhibited bruising characteristic of infanticide attacks by parrotlets (see Figs. S1-S3). The sex of attackers at 10 additional nests that were abandoned prior to infanticide attacks are not included.

	Count			Proportion		
**Attacker**	**Seen**	**Suspected**	**Both**	**Seen**	**Suspected**	**Both**
Male	14	63	77	0.58	0.66	0.65
Female	10	32	42	0.42	0.34	0.35
Total	24	95	119			

Table S2. Social status of green-rumped parrotlet individuals at 111 nests that were directly observed (Seen) committing infanticide attacks and those suspected of committing attacks. Observed attackers were individuals that were seen entering and/or emerging from nest boxes that contained parrotlet young or eggs. After the birds departed, checks of the nest contents by observers confirmed the killing or wounding of nestlings or eggs. Suspected attackers were individuals that were frequently observed attempting to enter nest boxes, perching on or near the box, and/or harassing the intact nesting pair or the widowed parent after a mate loss. We lacked sufficient evidence to determine the social status of attackers at 135 additional nests with eggs or chicks that were killed or wounded and exhibited bruising characteristic of infanticide attacks by parrotlets (see Figs. S1-S3). The status of attackers at 10 additional nests that were abandoned prior to infanticide attacks are not included. The social status of observed and suspected attackers (combined into 3 categories: male-female pairs, stepparents, and others) did not differ significantly (χ^2 = 0.091, df = 2, p = 0.955), so were combined for further analysis.

	**Count = 111**		**Proportion**	
**Attacker Social Status**	**Seen =18**	**Suspected = 93**	**Seen =18**	**Suspected = 93**
Male-Female Pair	10	55	0.556	0.591
Stepfather	5	22	0.278	0.237
Stepmother	1	1	0.056	0.011
Male-Male Pair	0	6	0.000	0.065
Unmated Male	2	9	0.111	0.097
Unmated Female	0	0	0.000	0.000

Table S3. Social status of green-rumped parrotlet individuals directly observed (Seen) committing infanticide attacks and those suspected of committing attacks at nests in relation to infanticide context: intact pairs (61 with status known out of 174 attacked nests), widows (41 out of 59 attacked nests) and widowers (9 out of 13 attacked nests). These data appear in aggregate form in Table S2 and are displayed in Figure 2A. See Table S2 legend for additional details.

Infanticide Context	Attacker Social Status	Seen	Suspected	Both	Prop
Intact Pairs	Male-Female Pair	5	41	46	0.754
	Stepfather	1	-	1	0.016
	Stepmother	-	-	-	0.000
	Male-Male Pair	0	6	6	0.098
	Unpaired Male	1	7	8	0.131
	Unpaired Female	0	0	0	0.000
	Totals	7	54	61	
Widowed Females	Male-Female Pair	3	9	12	0.293
	Stepfather	4	22	26	0.634
	Stepmother	-	-	0	0.000
	Male-Male Pair	0	0	0	0.000
	Unpaired Male	1	2	3	0.073
	Unpaired Female	0	0	0	0.000
	Totals	8	33	41	
Widowed Males	Male-Female Pair	1	5	6	0.667
	Stepfather	-	-	0	0.000
	Stepmother	2	1	3	0.333
	Male-Male Pair	0	0	0	0.000
	Unpaired Male	0	0	0	0.000
	Unpaired Female	0	0	0	0.000
	Totals	3	6	9	

Table S4. Comparison of logistic regression models for the probability of nest abandonment at 170 nests of widowed parents in relation to days after laying of the first egg and its quadratic form, the sex of the widowed parent (i.e., widowers versus widowers), whether the widowed parent re-paired with a potential stepparent, and whether an infanticide attack occurred. The full model contains all parameters except the quadratic terms for days after laying, which was les informative compared to the linear model (ΔAICc = 1.56). Presented are number of model parameters (*K*), Akaike's Information Criterion corrected for small sample size (AICc), the difference between the best model (bolded) and other competing models (ΔAICc), and the AICc weight (wt), which indicates the support for each model relative to others in the model set. See Figure 1 for the sample of nests in each category and Fig. 4E for visualization of the best model.

Model	*K*	Log likelihood	AICc	ΔAICc	AICc wt
**Days after laying + Infanticide attack**	**3**	**-75.518**	**157.2**	**0.00**	**0.736**
Full model	5	-74.458	159.3	2.10	0.257
Days after laying	2	-82.47	169.0	11.83	0.002
Infanticide attack	2	-82.54	169.2	11.98	0.002
Days after laying + Re-pair	3	-81.85	169.8	12.66	0.001
Days after laying + Days after laying^2	3	-82.21	170.6	13.39	0.001
Days after laying + Widowed parent sex	3	-82.36	170.9	13.69	0.001
Re-pair	2	-88.70	181.5	24.28	0.000
Widowed parent sex	2	-88.75	181.6	24.40	0.000

Supplemental Movie Legends
(due to PNAS' 10 mb limit for movies, clips had to be short)

Movie S1. Stepfather green-rumped parrotlet (#7920) attacks a brood of seven nestlings as the widow (A201) watches in foreground (Nest# 12B104A). All nestlings were repeatedly attacked by 7920, a 2-year-old who had yet to breed, over the following weeks, resulting in fatalities of the youngest nestling (24 October 2012). Six larger nestlings fledged successfully. The new pair did not go on to nest together.

Movie S2. A nonbreeding male green-rumped parrotlet (#8557) attacks nestlings of an intact breeding pair (Male X645, Female A216) (23 July 2019). Near the end of the movie, blood from a nestling can be seen flowing from a wound on the head to the bill of the adult. This one-year-old male (#8557) killed three young nestlings, but four nestlings eventually fledged successfully from this nest (Nest# 19B104A). Seeds are visible in the crops of several nestlings, so the parents had been providing food. Parents X645 and A216 had nested together the previous year, and they went on to nest again in 2019 after this nest attempt was completed. Black markings on the nestlings' heads were used for individual identification during a study of vocal development.

Movie S3. Continuing on in time from Movie S3, the nonbreeding Male #8557 attacks the bill and head of another small nestling in Nest# 19B104A.

Movie S4. Contest among multiple male green-rumped parrotlets harassing an intact breeding pair (15 July 2019) at Nest# 19B31A. The male parent can be seen at the entrance of the nest box and his mate at the top. A fight ensues.

Movie S5. Normal parental feeding behavior exhibited by an intact male-female parrotlet pair passing food by regurgitating seeds into the mouths of their nestlings at Nest# 19B105B (26 Oct 2019).
